# Workload, diagnostic work-up and treatment of urinary tract infections in adults during out-of-hours primary care: a retrospective cohort study

**DOI:** 10.1186/s12875-020-01305-8

**Published:** 2020-11-10

**Authors:** Michelle Spek, Jochen W. L. Cals, Guy J. Oudhuis, Paul H. M. Savelkoul, Eefje G. P. M. de Bont

**Affiliations:** 1 Department of Family Medicine, CAPHRI Care and Public Health Research Institute, Maastricht University Medical Centre, P.O. Box 616, Maastricht, MD 6200 The Netherlands; 2 Department of Medical Microbiology, CAPHRI Care and Public Health Research Institute, Maastricht University Medical Centre, Maastricht, the Netherlands

**Keywords:** Urinary tract infections, Out-of-hours primary care, General practice, Cultures, Antibiotics

## Abstract

**Background:**

Urinary tract infections (UTIs) are one of the most common infections in primary care. Previous research showed that GPs find it challenging to diagnose UTIs and frequently divert from guidelines leading to unwarranted antibiotic prescriptions and inefficient use of diagnostics such as urinary cultures. We hypothesise that management of UTIs during out-of-hours care may be extra challenging due to a higher workload and logistical issues regarding diagnostic work-up and obtaining results. We therefore aimed to study the workload, diagnostic work-up and treatment of UTIs during out-of-hours primary care.

**Methods:**

We performed a retrospective observational cohort study in which we analysed a full year (2018) of electronic patient records of two large Dutch GP out-of-hours centres. All adult patients with UTI symptoms were included in this study. Descriptive statistics and multivariate regression were used to analyse diagnostics and subsequent management.

**Results:**

A total of 5657 patients were included (78.9% female, mean age of 54 years), with an average of eight patients per day that contact a GP out-of-hours centre because of UTI symptoms. Urinary dipsticks were used in 87.5% of all patients visiting the out-of-hours centres with UTI symptoms. Strikingly, urinary cultures were only requested in 10.3% of patients in which urinary culture was indicated. Seventy-four percent of the patients received antibiotics. Seventy-nine percent of the patients with a negative nitrite test still received antibiotics. Remarkably, patients at risk of complications because of a UTI, such as men, received fewer antibiotic prescriptions.

**Conclusions:**

In total, 74% of the patients received antibiotics. 8 out of 10 patients still received an antibiotic prescription in case of a negative nitrite test, and 9 out of 10 patients with an indication did not receive a urine culture. In conclusion, we found that correctly diagnosing UTIs and prescribing antibiotics for UTIs is a challenge that needs major improvement, especially during out-of-hours GP care.

## Background

Urinary tract infections (UTIs) are one of the most common infections in primary care and are characterized by dysuria, frequency, and urgency for micturition [1–3]. However, these symptoms are not specific for UTIs. UTIs vary in severity from uncomplicated cystitis to prostatitis or even pyelonephritis [4]. Annually, around 58 out of 1000 inhabitants of the Netherlands are diagnosed with a cystitis by their general practitioner (GP) [5]. Incidence rates are higher in women (70 out of 1000), compared to men (10 out of 1000). Based on this, UTIs are the most common complaint in women in primary care.

Incorrect diagnosis has a considerable impact on antibiotic resistance in case of overdiagnosis, or patient’s health in case of underdiagnosis. There is increasing evidence in literature that recognizes the worldwide problem of emerging antibiotic resistance [6, 7]. In comparison to other European countries, antibiotic prescription rates in the Netherlands are relatively low [8], and consumption is more or less stable over time [9]. However, accurate UTI diagnostics and subsequent antibiotic management is still challenging.

The guideline of the Dutch College of General Practitioners on UTIs describes the diagnostic criteria and corresponding management for Dutch patients with a putative UTI [10, 11]. Most UTI management is based on patient symptoms and subsequent prescription of empirical antibiotics [6]. Physicians can also perform additional diagnostic tests such as urine dipstick, a urine dip slide or a urine culture [11, 12]. The most commonly used diagnostic tool, a urine dipstick, provides fast results and is used as point-of-care test. However, diagnostic accuracy is not adequate enough with a positive predictive value of 84% for the nitrite test and a positive predictive value of 91%, and negative predictive value of 76% for combination tests also including leukocytes and erythrocytes [13–15]. Although a number of uropathogens can give a negative result of the nitrite test, suggesting erroneously that the patient does not have an UTI, this is currently the most clear point-of-care cut-off test available. The reference standard, urine culture with antimicrobial susceptibility testing, takes two to 3 days and requires a microbiological laboratory [16]. We do however know there is discussion about this reference standard as well since cultures were negative in 20 to 30% of women with UTI symptoms, while a quantitative Polymerase Chain Reaction (qPCR) showed positive result for a uropathogen [17].

Suboptimal diagnostic procedures can lead to both over- and undertreatment of UTIs. Underdiagnoses of UTIs can lead to serious and potentially life-threatening complications like pyelonephritis or even urosepsis [4]. Furthermore, UTIs can lead to delirium, which is associated with acute care problems in elderly patients [18]. Overdiagnosis happens through empirical antibiotic treatment based on symptoms only or incorrect interpretation or classification of current diagnostic POC (Point-Of-Care) test results. Previous research has shown that 42% of antibiotic prescription for urinary tract complaints are not in line with guidelines [19]. This can partially be explained by unnecessary antibiotic prescriptions, but also by use of other antibiotics than recommended. This in turn contributes to the increase of antibiotic resistance, reduced treatment options and increasing health care costs [6, 7].

Because GPs and patients do not know each other during out-of-hours GP care and GPs do not have access to information on previous UTI episodes and urine cultures, we hypothesize that antibiotic prescription rates are even higher during out-of-hours care. One can assume that with acute onset of symptoms, and daytime GP practices only open during at best 30% of the 168 h in a week, a large proportion of UTI management happens at the out-of-hours care [20]. Earlier studies on UTI diagnostics have only focused on appropriateness of current diagnostic tests during daytime practice [4, 8, 20]. None of these studies analysed the diagnostic process of UTIs during out-of-hours care.

The specific objective of this study was therefore to examine the workload, diagnostic work-up and treatment of UTIs in healthy adults during out-of-hours primary care.

## Methods

We performed a retrospective observational cohort study. Data of electronic patient reports of two GP out-of-hours centres (located in Maastricht and Heerlen) in the province of Limburg, the Netherlands, during a full year (2018) were collected anonymously.

GP out-of-hours centres in the Netherlands are organised in large-scale cooperatives. These cooperatives cover primary care by rotating shifts of GPs during evenings, nights, and weekends. Telephone triage is done by triage nurses based on the Dutch triage standard (NTS), after which they decide if a patient has to visit the GP out-of-hours centres or not. The Dutch guidelines describe that patients with signs of tissue invasion or patients belonging to a risk group have to visit the out-of-hours GP centre. General practitioners with experience varying form 3 months to 30 years take care of patients visiting the GP out-of-hours centres. Seven GP out-of-hours centres in Limburg are responsible for urgent medical care during non-daytime hours [5]. The two above-mentioned out-of-hours centres are responsible for a population of 444,872 patients from both rural and urban areas and with different socio-economic status [21, 22]. The out-of-hours centre in Maastricht is responsible for a population of 179,426 patients and the out-of-hours centre in Heerlen is responsible for a population of 265,446 patients.

We included adult patients who visited one of the participating GP out-of-hours centres with UTI symptoms, defined as:

- ICPC codes: U01 (painful micturition), U02 (frequency), U05 (other micturition problems), U06 (hematuria), U07 (other urinary tract complaints), U70 (pyelonephritis), U71 (cystitis), Y73 (prostatitis);
- Patients with other ICPC codes with triage code ‘urinary tract problems’ or patients with terms ‘urine’ or ‘urinary tract infection’ in their medical report.

Patients with ICPC codes U70 (pyelonephritis), U71 (cystitis) or Y73 (prostatitis) were selected directly, while patients with other ICPC codes were selected if they had symptoms fitting with UTIs such as dysuria, frequency, or urgency for micturition. We therefore screened all patients with ICPC codes other than U70, U71 or Y73 manually and included patients only when the GP considered a UTI.

We excluded patients below the age of 18 years, patients with an indwelling bladder catheter, and patients with bladder malignancy. We defined the following groups as high risk UTI patients, according to the Dutch general practice guideline; men, pregnant women, patients using antibiotic prophylaxis for recurrent UTIs and patients with diabetes mellitus. The decision whether a urinary culture was appropriate and indicated was based on the Dutch general practice guideline. This guideline described that urinary cultures are indicated for high risk UTI patients.

The registered patient data consisted of information from telephone triage, given advice, consultation report, (working) diagnosis, ICPC code, treatment, and prescribed medication.

Primary outcomes were:

1. Number of contacts for UTI symptoms;

2. Number of diagnostic tests performed for UTI symptoms;

3. Antibiotic prescribing rate for UTI symptoms.

Secondary outcomes were:

1. Relationship between age and risk factors, and receiving an antibiotic prescription;

2. Urinary dipstick outcome related to antibiotic prescription.

Data analyses were performed using SPSS 25.0 (2018) and based on frequencies, descriptive statistics and binary logistic regression for antibiotic prescriptions (yes/no) as an independent outcome.

Relationships between covariates (age, risk factor for complications, outcome of urinary dipstick) and antibiotic prescription rates were analysed using multivariate logistic regression analyses. To determine which factors influence antibiotic prescription, we analysed all patients in our database except for the patients who were referred to the emergency department and the patients who had already received an antibiotic prescription from their own GP.

## Results

### Workload

In total, 5657 patients contacted the out-of-hours GP centre because of UTI symptoms during the study period. This corresponds with an average of eight patients per day. Most patients were women (78.9%) with a mean age of 54 years (range 18–104 years).

**Fig. 1:**
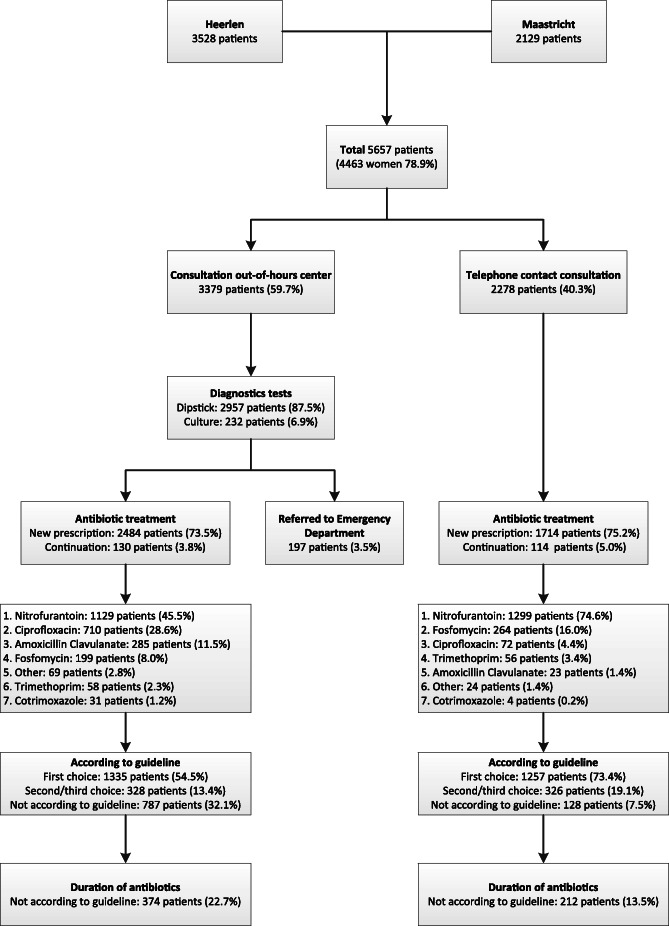
Flowchart showing the workload, diagnostics and management within patients that contacted GP out-of-hours care because of urinary tract infection symptoms

Figure 1 shows an overview of the diagnostic work-up and management of these patients. As shown in this figure, 59.7% of the patients visited the out-of-hours centre for a consultation or at least a dipstick, compared to 40.3% of the patients that only had a telephone contact. Patients visiting the out-of-hours centre were more often patients with one or risk factors (37.5%) compared to patients that had telephone contact (14.7%). Furthermore, patients with signs of tissue invasion during telephone triage visited the out-of-hours centre more often (18.1% versus 2.7%). No more than 3.5% of the patients visiting the out-of-hours centre were referred to the emergency department after consultation.

### Diagnostic work-up

Two diagnostic tests for UTIs were used at the out-of-hours GP centres: the urinary dipstick and urinary culture. The dipstick was used in 87.5% of all patients that visited the out-of-hours GP centre because of UTI symptoms. However, despite this test, antibiotic prescription rates are the same for patients that visited the out-of-hours GP centre and patient that had telephone contact consultation only.

Table 1 shows the amount of ordered cultures for the group with an indication for a culture and the group without an indication.

**Table 1:** Urine cultures in patients at the out-of-hours GP centres

	Indication (%)	No indication (%)
Total number of patients	1552 (29.8)	3663 (70.2)
Culture not ordered	1392 (89.7)	3526 (96.3)
Culture ordered	127 (8.2)	102 (2.8)
Referred to own GP	22 (1.4)	22 (0.6)
Ordered by own GP	13 (0.7)	11 (0.4)

Patients referred to the emergency department or patients who already received antibiotics from their own GP were excluded from this analysis

In total, 229 cultures were ordered. 96.3% of the patients without an indication did not receive a urinary culture. However, almost half of the cultures that were ordered were for patients without an indication for a urinary culture. Furthermore, 89.7% of all patients who had an indication for a urinary culture did not receive a urine culture at the out-of-hours GP centre.

### Treatment

Of the patients that contacted or visited the out-of-hours GP centre 74.2% received antibiotics. Of these antibiotic prescriptions 40.8% were given after telephonic contact without a physical consultation at the out-of-hours centre. A small proportion of the patients (3.8% of the consultation group and 5.0% of the telephonic consultation group) already had antibiotics prescribed by their own GP leading to no new antibiotic prescription in these patients.

An important finding was non-accordance to the guideline resulting in prescription of antibiotics that are not in line with the guidelines in more than 1 out of 5 patients. Furthermore, antibiotic prescription was more in line with the guidelines after telephone contact consultation compared to a consultation at the out-of-hours GP centre. Besides, the duration of antibiotics is not in line with the guidelines in more than 20% of the patients that visited the out-of-hours GP centre. Some of these patients received a too long prescription, while the prescription was too short in other patients.

Age did not significantly influence antibiotic prescription rates (median age 52 years non-antibiotic group versus 54 years in antibiotic group).

Table 2 presents the results of the multivariate logistic regression analyses studying which risk factors influence antibiotic prescription.

**Table 2:** Influence of risk factors on antibiotic prescription

	n (%)	Antibiotic prescription (%)	Unadjusted OR (95% CI)	Adjusted OR(95% CI)
Total	5215 (100)	4195 (80.4)	–	–
Low-risk for complications	3819 (73.2)	3299 (86.4)	–	–
High-risk (≥1 risk factor)	1396 (26.8)	896 (64.2)	0.3 (0.2–0.3)*	0.4 (0.3–0.5)*
*Male gender*	1030 (19.8)	630 (61.2)	0.3 (0.2–0.3)*	0.4 (0.3–0.5)*
*DM*	282 (5.4)	229 (81.2)	1.1 (0.8–1.4)	1.0 (0.6–1.6)
*Pregnancy*	161 (3.1)	86 (53.4)	0.3 (0.2–0.4)*	0.3 (0.2–0.5)*
*Previous antibiotic prophylaxis*	16 (0.3)	15 (93.8)	3.7 (0.5–27.7)	0.6 (0.1–5.7)

*Significant differences, *p* < 0.05

*DM* diabetes mellitus

As shown in the table above, a negative correlation was found between the risk factors: gender (male) and pregnancy, and antibiotic prescription. Antibiotic prescription rates appeared to be unaffected by diabetes mellitus and the use of antibiotic prophylaxis for recurrent UTIs.

Almost all patients (98.1%) received antibiotics in case of a positive dipstick for nitrite. However, in case of a negative dipstick for nitrite, 78.8% the patients received antibiotics as well. A positive dipstick for nitrite was a predictor for antibiotic prescription with an adjusted OR (corrected for age, gender, risk factors, and results of leukocytes and erythrocytes in the dipstick) of 14.2 (95% CI.: 8.1–24.8).

In case of a negative dipstick for leukocytes, 64.7% of the patients received antibiotics. Leukocytes in a dipstick test was a predictor for antibiotic prescription with an adjusted OR (corrected for age, gender, risk factors, and results of nitrite and erythrocytes in the dipstick) of 2.0 (95% CI.: 1.8–2.2).

In case of a negative dipstick for erythrocytes, 74.7% of the patients received antibiotics. Erythrocytes in a dipstick test was a predictor for antibiotic prescription with an adjusted OR (corrected for age, gender, risk factors, and results of leukocytes and nitrite in the dipstick) of 1.2 (1.1–1.3).

## Discussion

An average of eight patients per day contacted the GP out-of-hours centre because of UTI symptoms. In total, 74% of the patients contacting the GP out-of-hours centre because of UTI symptoms received antibiotics. Urinary dipstick was used in almost all patients (87.5%) that visited the out-of-hours GP centre with UTI symptoms, but 8 out of 10 patients still received an antibiotic prescription in case of a negative nitrite test. Furthermore, 9 out of 10 patients with an indication did not receive a urine culture, while 44.5% of all cultures ordered at the out-of-hours GP centre were for patients without an indication for a urinary culture.

In agreement with our study, studies from other countries and studies performed in regular GP practices have shown that chosen diagnostics, in most cases, do not correspond to guidelines [8, 22]. A recent Dutch study during office hours has shown that recommendations for ordering urinary cultures in risk groups, such as pregnant women, and not using urinary cultures in non-risk groups are often not applied in daily practice [4]. This is striking while these criteria are clearly described in the guidelines and GPs have access to full medical reports of their own patients.

As hypothesised, our study showed an even lower number of correctly ordered cultures of one out of ten patients [4, 8, 23]. This is far below the one out of three that was found in an earlier study during office hours [4]. A possible explanation for this difference could be that GPs could feel less responsible for urine cultures of patients if they are not confronted with eventual problems during follow-up, because patients will visit their own GP for this. Another reason for GPs not performing urine cultures at the out-of-hours care could be the inability to safely follow-up the result, while they are responsible for this when they request the culture. A third explanation for this was shown in our database, where some GPs said that patients should visit their own GP for a culture while this is not possible during out-of-our care. This implicates that GPs are either unaware of the fact that cultures are possible during out-of-hours care or experience (time) barriers to perform a culture during out-of-hours care. This while patients consulting out-of-hours care have a higher risk of complications and efficient use of diagnostics and correct use of antibiotics might be even more important. Future research is needed to clarify this matter and to study whether improved (point-of-care) diagnostics can improve GPs’ management of UTIs. A study at the out-of-hours care in Norway showed promising results by using a diagnostic algorithm instead of a consult with a GP [24]. This could be interesting in the Netherlands as well because our study showed that antibiotic prescription is least in accordance to the guideline in patients visiting the out-of-hours GP centre. A further study with more focus on the use of such an algorithm in the Netherlands is therefore suggested.

The number of antibiotic prescriptions is similar to those found by another study in the Netherlands [10]. However, adherence to the guidelines observed in this investigation is far below that observed in another Dutch population [4]. We believe this could be explained by a difference in setting, out-of-hours care, instead of during daytime practice. During out-of-hours care, GPs generally do not have access to patient’s previous urine culture results or prior UTI symptoms because of privacy reasons and lack of a shared electronic patient file or treatment relationship during daily practices. Therefore, it is difficult to determine if a patient is part of a risk group or not. Due to lack of this information in medical reports, GPs determine if a patient belongs to a risk group on subjective anamnestic information only. Furthermore, our population could be different from the population analysed by Ganzeboom et al. [4], since they only included three ICPC codes specific for UTI, prostatitis or pyelonephritis. However, these findings are still alarming and future research is therefore needed.

Another remarkable aspect is the fact that two risk factors, male gender and pregnancy, seem to lead to fewer antibiotic prescriptions. This unexpected result was not described in literature before, we do however know that symptoms might be less specific in males [25, 26]. Less specific complaints resulting in a broad differential diagnosis could be a major factor causing the low number of antibiotic prescriptions in these risk groups. However, the same result was found after a subgroup analysis of cases with ICPC codes specific for cystitis, prostatitis and pyelonephritis only (data not shown).

This is the first study to provide an insight into the workload, diagnostic work-up and management of UTIs at GP out-of-hours centres during a full year. The most important strengths of this study were the number of participants (5657 in total) and the fact that the data of these patients were routinely collected during normal GP out-of-hours care. GPs and triage nurses were not aware of the fact that we were studying their management and could therefore not adapt their behaviour to desirable outcomes.

### Limitations

In observational studies, there is a potential for bias from missing data. As discussed earlier, decision making could be difficult for GPs when they do not have full access to a patient’s medical report as in daytime practice. This could lead to an underestimation of risk factors that are important to choose the right antibiotic for a patient. However, it is also possible that information is missing in the report at the out-of-hours GP centre when a GP has actually asked this but did not write it down properly in the medical report. Furthermore, the written content in medical reports is dependent on the interpretation of the GPs or triage nurses. This could have influenced our results, since we might have categorized some patients in the wrong risk group and compared the choice of antibiotic prescription with the wrong guideline. On the other hand, we believe that the benefits of our design outweigh these limitations.

## Conclusions

An average of eight patients per day contacted their GP out-of-hours centre because of UTI symptoms, of which 74% received antibiotics. Urinary dipstick was used in almost all patients (87.5%) that visited the out-of-hours GP centre with UTI symptoms, but 8 out of 10 patients still received an antibiotic prescription in case of a negative nitrite test. Furthermore, 9 out of 10 patients with an indication did not receive a urine culture, while 44.5% of all cultures ordered at the out-of-hours GP centre were for patients without an indication for a urinary culture.

Research questions are rising why GPs decide to deviate from UTI guidelines and if GPs are even aware of this fact. Another study showed that a therapy suggestion list in combination with limited availability of ciprofloxacin reduced prescription rates for this antibiotic in cases when other antibiotics are preferred, based on national guidelines [27]. Although UTIs are one of the most common infections, UTI diagnostics and management is still challenging and needs major improvement, especially during out-of-hours GP care.

## Data Availability

The anonymised data used and analysed during the current study are available from the corresponding author on reasonable request.

## Abbreviations

AB: Antibiotic
DM: Diabetes mellitus
GP: General practitioner
ICPC: International Classification of Primary Care
OR: Odds Ratio
POC: Point-Of-Care
UTI: Urinary tract infection
